# Bridgewater Treatises

**Published:** 1836-07-01

**Authors:** 


### THE BRIDGEWATER TREATISES.

Chemistry, Meteorology, and the Function of Digestion, considered with reference to Natural Theology. By *William Prout*, M.D. F.R.S. &c. &c. London, 1834.

[Concluded from No. 48.]

We noticed the two first divisions of the Bridgewater Treatise before us, in the last number of this Journal. The principal arguments in support of the wisdom and goodness of a Deity, drawn by Dr. Prout from the facts and phenomena of chemistry and meteorology, were laid before our readers. We deferred the consideration of the concluding portion of the volume, which is occupied with the chemistry of organization, and particularly with the chemical process of assimilation. This consumes about 140 pages, and we shall proceed to give a sketch of Dr. Prout's opinions and reasonings upon the subject.

In discussing chemistry and meteorology, inorganic substances formed the chief objects of inquiry. We are now to be occupied with organized beings, and with the means and the processes by which their organization is effected.

Organic bodies are, independently of those subtle agencies denominated vital, chemical compounds. Both animal and vegetable, unlike as they appear and insensibly blended as they really are, consist of certain elementary bodies, termed the ultimate elements of organization—and of secondary compounds, named its immediate or proximate elements.

"Vegetable substances," observes Dr. Prout, "in general, contain essentially no more than three elements, *Hydrogen, Carbon,* and *Oxygen;* while animal substances usually involve a fourth. *Azote.* Yet there are many vegetable substances, of whose composition, azote forms a considerable part; while certain animal substances are entirely wanting in that principle. It is obvious, therefore, that the mere chemical composition of a substance, at least its essentially consisting of three or of four of these elements, will not enable us to determine whether it be vegetable or animal; and that, in many instances, when this point happens to be doubtful or unknown, we must have other data before we can form a conclusion. Besides these four elements, of which all organic substances are essentially compounds; other principles generally enter into their composition. These other principles are in very minute quantity, and are not so essential to the actual existence of organic substances, as the four constituent elements above named; yet, however minute the quantity, the influence of these other principles seems to be most important; they are, *Sulfur, Phosphorus, Chlorine, Fluorine, Iron, Potassium, Sodium, Calcium, Magnesium,* and probably more besides. These principles have, by most chemists, been deemed extraneous, or foreign to organized bodies; but we shall presently show, that there is good reason to believe, that the office of such additional principles, though different from that of the constituent elements, is nevertheless most remarkable. These four elements, along with the additional principles, are, in the present state of our knowledge, alike denominated, *The Ultimate Elements* of organized bodies; but hydrogen, carbon, oxygen, and azote, may be termed, for the sake of distinction, the *essential* elements; and sulfur, phosphorus, &c. the *incidental* elements of such bodies. The combinations of these ultimate elements with one another, according to certain laws, produce what are denominated the *Immediate*, or *Proximate Elements* of organized bodies. Of these proximate elements, *Sugar, Oil, Albumen,* &c. are familiar examples." 417.

Perhaps, continues Dr. Prout, no substance actually forming a constituent part of a *living* plant or animal, is so pure as to assume a regularly crystallized form. All solid organized substances are, therefore, more or less rounded in their form, and any thing but crystallized in their interior. Organized fluids are very heterogeneous in their composition, although the basis of nearly every one is water.

Dr. Prout arranges organized bodies in two general classes:—those which, though they do not crystallize while in the living plant or animal, can yet, by various processes, be so far separated from extraneous matters, as to be obtained in a state of purity, and thus be made to assume the crystallized form; and those which cannot under any circumstances be made to crystallize.

Sugar and vinegar are instanced by Dr. Prout as members of the crystallizable family. Both contain oxygen and hydrogen, in the proportions requisite for the production of water and carbon, and it is remarkable the composition of the two should be so similar.

The uncrystallizable proximate elements alluded to by Dr. Prout, are starch and lignin. Starch consists of a number of granules. Its composition is so analogous to that of sugar, that chemistry detects but little difference in the proportions of the constituent parts. Lignin consists of equal weights of water and of carbon, the same elements, again, almost in the same proportions, producing a substance widely different in its physical qualities. The following Table exhibits at a glance the preceding points of similarity and distinction between sugar, acetic acid, starch, and lignin.

TABLE.

| *Substances Crystallizable.* | | | *Substances* not *Crystallizable.* | | |
|---|---|---|---|---|---|
| | Carbon. | Water. | | Carbon. | W[illegible] |
| SUGAR, from Starch........ | 36·20 | 63·80 | STARCH, Arrow-root in its ordinary state .... | 36·4 | [illegible] |
| from Honey........ | 36·36 | 63·63 | from Wheat, in its ordinary state ... | 37·5 | [illegible] |
| East India Moist.... | 40·88 | 59·12 | ditto, ditto, dried at 212° ........ | 42·8 | [illegible] |
| Beet-root and Maple, | 42·10 | 57·90 | | | |
| English Refined .... | 41·5 to 42·5 | 58·5 to 57·5 | | | |
| Pure Sugar Candy .. | 42·85 | 57·15 | | | |
| ACETIC ACID.............. | 47·05 | 52·95 | ........................ | | |
| ........................ | | | LIGNIN, in its ordinary state of dryness ...... | 42·7 | [illegible] |
| | | | from Willow, dried at 212° ......... | 49·8 | [illegible] |
| | | | from Box, ditto .... | 50 | [illegible] |

By organic agency these principles are all easily convertible. By artificial processes we can change starch and wood either into sugar or vinegar—wood into a sort of starch—and sugar into vinegar; but we are unable to reverse the process and convert vinegar into sugar, or starch into wood.

The question naturally arises—how is it that substances, so nearly allied in composition, should exhibit such different sensible properties? To answer this question, Dr. Prout indulges in some theoretical considerations, which leave us, however, as much in the dark as we previously were, with respect to the actual cause of the phenomena. Indeed Dr. Prout's own remark—that these are ultimate facts--is the most satisfactory reply that can be offered. Such ultimate facts are withdrawn from the dominion of reasoning, and, perhaps, an abrupt confession of ignorance is more philosophical and more correct than attempts to explain what we cannot comprehend.

But although we are unable to understand the mysterious operation of the laws of chemical and vital combination, we may investigate the attendant circumstances, and render ourselves familiar with those facts which are within the scope of observation. Dr. Prout remarks that organized bodies may be viewed as differing in the composition of their essential—or in that of their incidental elements. Thus, sugar is composed of 42.85 per cent. of carbon, and the rest water: while vinegar consists of 47.05 per cent. carbon, and the residue water. Here the difference in the essential elements is attended with a most remarkable difference of properties. But the essential elements of sugar and starch are almost the same, and the difference of composition is in the starch containing some incidental bodies, from which sugar is free. Dr. Prout supposes that these incidental bodies are interposed between the constituent molecules of the substance which are in a state of self-repulsion. But this is so conjectural that we may pass it by. We fear that this theory of the phenomena of vitality, though more chemical than many of its predecessors, adds no more than they have done to the sum of our knowledge on the subject—a sum which amounts to little more than absolute and hopeless ignorance.

Between organized and inorganic matter, there is a visible distinction. The metaphysical atheists, however they may envelop their own reasoning faculties in an impenetrable fog of abstract ideas or words, have never been enabled to extinguish the common sense of mankind, and to prevent it from seeing, feeling, and acknowledging the difference between living matter and dead. If organization consists simply in the chemical combination of certain elementary substances, and if life be "the sum of organization," it follows of course, that the combinations of elements constitute life, which is no more than the result of chemical action. Such is the close reasoning of some modern philosophers, who have established for themselves an enviable reputation, by the promulgation of such ridiculous fallacies.

The whole argument is based on an assumption which they cannot prove—an assumption, that it is the combination of the elements which gives rise to the phenomena of life. Let us look at this.

If two or more elementary bodies combine, a product is the consequence, possessed of certain characteristic properties, and accompanied, in its formation, by certain phenomena. Those phenomena always occurring under similar circumstances, constitute the evidence, and the product, the effect of what we denominate chemical action. If two volumes of hydrogen and one of oxygen are mixed and detonated, the result is water; and, no disturbing agents interfering, that result always follows the same antecedents.

It is to such an action as this that vitality has been compared. Yet observe how foolishly. We ascertain by chemical analysis, that an organic substance is made up of certain elementary materials in certain constant proportions. So far it displays the properties of every chemical compound. We endeavour artificially to reproduce it—we bring its elements together—we apply every circumstance favorable to their combination—but, never yet, save in mythology or fable, has any organized being or the smallest atom of a substance of organic character sprung from the atheistical still.

The assumption, then, that vitality is the product of elementary action, is founded on an analogy, which is demonstrably false. Chemical compounds may be recomposed—organic compounds cannot. Even Nature, that is, Nature's God, does not exert the power of *producing* living things, by the mere conjunction of their elements. Chemical compounds are constantly destroyed and as constantly re-formed. The very elements of organization are continually discarded and resumed. But life is not so resolved and reconstructed, and the Deity has shewn how distinct it is from chemical action by the solicitude with which he has contrived the process of organic generation. Even the researches on the production of the infusoria have left the dogma—omne vivum à vivo, unshaken.

Analogy, even when it presents no apparent imperfection, can seldom be received as a conclusive argument. It may form a part, an important part of cumulative reasoning, but, by persons of exact minds it will not be permitted to do more. What then shall we say of analogy palpably defective—what shall we think of reasoners, who, affecting to despise the prejudices of mankind, and to distrust the principles that have influenced its belief, employ a logic more inexact than that which they would overthrow, and build *their* conclusions on premises so flimsy that a school-boy could confute them?

It may be said that the materialists do not contend for the operation of *ordinary* chemical action, in the production of the phenomena of life. But if it be not ordinary chemical action, what will these gentlemen tell us that it is? Their boasted philosophy will leave them where it picked them up, and after all their doubtings they must come to the point of common sense from which they started; and own that this something, which is not chemical, which is not mechanical, which differs in many important respects from any agency common to inorganic matter, is something sui generis—in short is—life.

The organic agent, or agents, whatever it or they may be, controul and modify the laws of matter. They modify them to a certain extent—to that extent conducive to the constitution of organized beings, such as we find it to be. Whether among the innumerable possibilities of combination there might not have been some more perfect than those we are actually acquainted with, is not the present question. Our business is with Nature as she is, and not as she might otherwise have been. Dr. Prout, to whom we now return, enquires what the modes of operation of the organic agents are.

"In the first place," says Dr. Prout, "with regard to what *cannot* be effected by organic agency, we may observe, that no organic agent has the power either of *creating* material elements, or of *changing* one such element into another.

By element, it may be right to premise, is here meant, a principle that is not made up of others; and which, consequently, possesses an absolute and independent existence. Whether one, or more, such elements exist, it is not now our object to enquire. The astonishing discoveries of modern chemistry have shewn, that many of those substances, formerly considered as elements, are, in fact, compounds; and as the science of chemistry is still progressive, it is probable that, with the enlargement of its boundaries, there will be a still further diminution of the number of those substances which are, as yet, held to be simple. Admitting, however, for the sake of argument, that elementary principles do exist, of such immutable character as has been supposed; from the nature of organic beings, at least of all animals, it is impossible to conceive that they possess the power either of creating or of altering these elementary principles. For no organized being has an independent existence, but all animals derive their support from previous organization, which might be otherwise, did they possess a creating power; nor can they be nourished by all substances indiscriminately, as they ought to be, were they possessed of a transmuting power. Yet, while it is thus denied that organized beings possess the power, either to create or to change, in the strict acceptation of these terms; it has been admitted to be exceedingly probable, that the organic agent is, within certain limits, qualified to compose and decompose many substances which are now viewed as elements; and that the organic agent does thus apparently form and transmute these imagined elements. But to enter further, in this place, on the elucidation of these obscurities would be foreign to our present purpose." 432.

The organic agent has not the power of combining elements in such a manner, that the properties of the resulting compound shall be different from those of a compound, formed from the same elements similarly combined by any other agent. Of course there are limitations to the powers of vitality, and this is one. If the organic agent combines oxygen and hydrogen in the proportions in which they are combined in water, only water can result. Such is the position of Dr. Prout. We give it as it stands, yet we may observe that our acquaintance with the facts of organization is perhaps too limited to enable us to receive it with implicit confidence. There is no absurdity in supposing that combinations, the result of ordinary chemical affinities, may be modified by other powers; or, in imagining that, under peculiar circumstances, vitality may exert that influence. This is not a question capable of being determined by reasoning, but only by observation of the fact. Indeed, so far as the very imperfect knowledge we possess on the powers of organization goes, it rather seems to shew that they do modify chemical action. If we find for example that the living principle, out of certain proportions of elements forms sugar, and that nothing save the living principle can combine those elements in such a way as to produce that compound, it is a priori evidence in favour of the belief that vitality does disturb affinities, and alter their results. It is obvious that, whatever our speculative notions on the subject may be, our acquaintance with the actual operations of nature is exceedingly limited and vague.

The means by which organic agents effect the required purposes, are divided by Dr. Prout into two kinds:—those which are dependent on peculiarity of composition and of structure; and those by which this peculiarity of composition and of structure is produced.

Although the ultimate elements of organization are not numerous, and are generally diffused through the various parts of each organic being, yet the proximate principles formed by the organic agent out of those elements, are more exclusively appropriated to particular parts and functions. Thus the woody fibre of plants is always formed of lignin, and never of resin or albumen. Peculiarities of structure, therefore, almost invariably indicate peculiarities of composition—and similarity of structure, identity, or, at all events, similarity of composition.

2. The means by which that *peculiarity of composition and of structure is produced,* which is so remarkable in all organic substances, like the results themselves, are quite peculiar; and bear little or no resemblance to any artificial process of chemistry. For example, we have not, in artificial chemistry, any control over individual molecules; but are obliged to direct our operations on a mass, formed of a large collection of molecules. The organic agent, on the contrary, having an apparatus of extreme minuteness, is enabled to operate on each individual molecule separately; and thus, according to the object designed, to exclude some molecules, and to bring others into contact. Dr. Prout proceeds with some ingenious, but fanciful suppositions on the modes in which these molecules, and molecular powers act. We fear, however, that all is too uncertain to detain the exact reasoner. Dr. Prout adverts to the notion of spontaneous development of organization maintained by some distinguished French philosophers. Whether distinguished or not in other ways, they have been eminently distinguished for absurdity in this. Even Voltaire held up this folly to ridicule, and in one of his Contes en Vers, he introduces a Sage of this Harlequin school, developing an oyster into a man. One reply is sufficient for this theory. Can its advocates shew us the fact? If it is a fact they ought to shew it, and they could do so. This spontaneous development will probably be exhibited when the transmutation of metals is effected.

Dr. Prout concludes the chapter with some remarks on the powers displayed by the Creator, in uniting the ultimate elements of bodies, with the vital agents. All is indeed wonderful and incomprehensible here. But it is a melancholy truth, that what excites devotion and awe in a religious mind, hardens and confirms scepticism in the sceptic. What is incomprehensible is no longer a matter of demonstration or of argument, and the sullen atheist, confident in the reason that he abuses and debases, shuts his ear to the Hosanna of humble and of grateful adoration.

One idea of Dr. Prout's may be glanced at for its singularity. Amidst the wonders of Creation, he observes, it is perhaps difficult to say what is most wonderful; but we have often thought, that the Deity has displayed a greater stretch of power, in accommodating to such an extraordinary variety of changes, a material so unpromising and so refractory as charcoal, and in finally uniting it with the human mind; than was requisite for the creation of the human mind itself. To Him, however, all things are alike easy of accomplishment; and He, doubtless, has willed these and other proofs of His omnipotence, in order to convince us of this truth,—that the Creator of the mind, could alone have created the matter with which the mind is associated! When we look at the elements of which man is made, and when we turn from the material composition of the brain to its intellectual and moral operations, we are compelled to own how utterly inadequate all reasoning is to the explanation or the comprehension of this mystery. The vain sophist is to be pitied, who can satisfy himself that the water and the carbon of which his cerebrum is manufactured, are sufficient to produce by their chemical combination, even the poor intellect that *he* evinces.

The Second Chapter is occupied with the modes of nutrition—and a sketch of the alimentary apparatus, and of alimentary substances, in plants and in animals.

Nutrition, of course, presupposes the assumption, by the body nourished, of extraneous matters. The nutrition of animals and plants is effected, as might be expected, by means of a different description.

### 1. *Of the Nutrition of Plants.*

"A few forms of tissue," says Professor Lindley, and our author quotes his words, "interwoven horizontally and perpendicularly, constitute a *stem;* the development, by the first shoot that the seed produces, of buds which grow upon the same plan as the first shoot itself, and a constant succession of the same phenomenon, causes an increase in the length and breadth of the plant; an expansion of the bark into a *leaf,* within which ramify veins proceeding from the seat of nutritive matter in the new shoot, the provision of air-passages in its substance, and of evaporating pores on its surface, enables the crude fluid sent from the roots to be elaborated and digested until it becomes the *peculiar secretion* of the species: the contraction of the branch and its leaves forms a *flower;* the disintegration of the internal tissue of a *petal* forms an *anther;* the folding inwards of a leaf is sufficient to constitute a *pistillum;* and finally, the gorging of the pistillum with fluid which it cannot part with, causes the production of a *fruit*." 445.

The sap of plants consists of water, mucilage, and sugar, with some minute portions of other matters, principally saline. Though some moisture is absorbed by the leaves, the principal portion enters by the roots, and particularly by the fibrous parts termed *spongioles*. It seems to be established that the roots, besides imbibing nourishment, perform also an excretory office; and that in the soil in which plants grow, there are deposited by the roots certain matters of an excrementitious nature, injurious to the plants from which they have been separated; and which, therefore, cannot be absorbed again till they have undergone decomposition. Such excreted matters have been adduced as the reason, why a soil becomes so much deteriorated by any one species of plant having long grown in it, that it will not support other individuals of the same species: whence the necessity of a rotation of crops.

The quantity of water or sap in some plants is very great, as is the force with which, on the approach of warm weather in our climate, or of the rainy season within the Tropics, it is determined upwards. The composition of the sap varies in different parts of the same plant; it is little more than water in the roots, but as it ascends it acquires more and more of saccharine and other matters.

When the sap begins to rise, the leaves begin to be developed. From the leaves more particularly, a copious evaporation of the watery portions of the sap takes place. The more solid matters, dissolved in a less proportion of water, after undergoing further changes, probably for the most part in the leaves, are returned to be deposited in other parts of the plant.

"It seems now to be generally admitted, that one part of the food of plants is the matter extracted from the soil; and that this matter is taken up with the watery portion of the sap above-mentioned. It seems also to be admitted, that carbonic acid gas is in some way indispensable to vegetation ; 'for it has been ascertained that, feed plants as you will, they will neither grow nor live, whether you offer them oxygen, hydrogen, azote, or any other gaseous or fluid principle, unless carbonic acid is present.' Like the other nutritious matters, this carbonic acid is partly taken up by the roots; but, under certain circumstances, it is also separated from the air, and absorbed by the leaves. The circumstances under which this absorption, or rather decomposition, of carbonic acid, by the leaves takes place, are most curious and important. They are understood to be as follows :

During the day, and particularly during sunshine, the leaves of plants have the power of abstracting the carbonic acid from the atmosphere. The carbon of the acid, and perhaps also a little of its oxygen combine with the plant ; while the greater part of the oxygen remains, and is diffused through the atmosphere in a gaseous state. During the night, on the contrary, or in the shade, plants, in general, convert a portion of the oxygen of the atmosphere into carbonic acid ; but the quantity thus converted, is less than that separated from the carbonic acid which they decompose, under the influence of the solar light. At the same time with this formation of carbonic acid by plants during the night, they are said also to absorb from the atmosphere a certain portion of oxygen ; to replace that which had been given off, during exposure to sunshine, on the preceding day. Plants absorb carbon as long as they are exposed to the light; during the season, therefore, when the day is long and the night is short, plants give off much less carbon than they absorb. This excess of the absorption of carbon is probably one reason why, in the Polar latitudes, the progress of vegetation is so rapid. By a beautiful provision of nature, in the course of the short summer of a few weeks, but of unvarying light, plants, in these latitudes, go through all the changes which in hotter climates require many months.

These phenomena of gaseous absorption and secretion in the leaves of plants, seem to be produced by a portion of the leaf peculiarly organized, and situated immediately under its external covering or epidermis. Professor Burnet has lately explained these phenomena, by referring them to the respiration and digestion of plants. The process of respiration in plants, is supposed to be continual; and to be accompanied, as in animals, by the formation and emission of carbonic acid gas. While digestion, which consists in the decomposition of carbonic acid gas, takes place only during the exposure of plants to the influence of the light—the carbon of the carbonic acid being separated from the oxygen, and absorbed. Hence a plant exposed to sunshine purifies the air, by digesting the carbonic acid, the carbon of which it appropriates ; while it sets the oxygen free. In the dark, on the contrary, digestion ceases, but respiration continues ; and carbonic acid gas is thus accumulated in the surrounding atmosphere." 449.

The peculiar principles of plants are very numerous, but admit, Dr. Prout conceives, of classification under three heads ;—the saccharine—the oily—and the acid. Some vegetable principles contain azote, and perhaps other elements.

2.—*Of the Nutrition of Animals.*

We may pass by Dr. Prout's description of the organs of digestion in man and in animals. It is a lucid sketch, well adapted for the general reader, to whom it is addressed. Dr. Prout makes an observation, in which we cannot help thinking there is much truth, although the opinion has been hotly denounced by the transcendental vitalists. The observation is this—that the organic or vital agents operating in effecting the process of digestion, are probably in man, as in other animals, not very far removed from the agencies of mere inorganic matter. When we look at the process of chymification and defæcation, we must admit that they resemble very closely those of a chemical character. It is true that the secretions which are poured upon the aliment, and are operative in effecting the changes in question, are under the control of the nervous influence, and it is most probable that nervous influence, here as elsewhere, is one of the higher attributes of organization; yet, even with this abatement, the process of assimilation in plants, and, indeed in animals, may be regarded as the initiative of organic agencies and actions, the connecting link between the living and the dead.

The consideration of alimentary substances next occupies our author. As his researches on this point have been long before the public, we shall touch on them but lightly. But to many of our older readers, who cannot be acquainted with the contents of the later works on physiology, they will probably be novel.

It may be stated, as a general rule, that animals seem destined by nature to feed on those below them in the scale of organization. Of course this must be regarded as only the expression of the most general law that the case admits. The lower animals are endowed with organs which enable them to digest the grasses and the vegetables most difficult of assimilation; and, having done this, they become the food of other and of higher animals, whose organs are not fitted for the digestion of those simple elementary aliments.

The compatibility of this scheme of carnivorous feeders, with the notions we possess of perfect benevolence on the part of the Creator, has been frequently denied. It must be owned that the defence has never been satisfactory as an argument. The following may be considered as the principal reasoning employed by the advocates of the system of preying on each other, that distinguishes the animal creation.

> "If organized beings did not prey on each other, their remains would, in time, accumulate in such quantity, as to be nearly incompatible with life; certainly with animal life in its most perfect condition, as it is at present known to us. But by the arrangement that animals are food to each other, not only is an opportunity afforded, for the existence of a greater number of animals, and of a greater variety among them; but the obtrusion of the bodies of animals, in whom life has become extinct, is entirely prevented: nor is the removal of the dead animal matter the only good accomplished, but many other important results are obtained." 472.

It is a very imperfect defence of the wisdom and goodness of the Creator, to urge that he has deemed it necessary to correct the redundancy of animal life, the result of his own laws, by wholesale cruelty. Similar reasoning justifies infanticide in China—proves, if it proves any thing, either the unskilfulness or the malevolence of the Divinity. The truth is, that the existence of this physical, as of many moral evils, is a natural fact that we must admit, but cannot understand. When we look around us, and see other and mightier worlds, and when, reasoning from what we do see to what is beyond our sphere of vision, we conjecture that there may be systems of worlds besides, we are compelled to feel our own insignificance, and to admit how inadequate both our minds and our knowledge are, to enable us to comprehend our position in the Universe. Of that Creation we form a proportion so minute, that what seems to be partial evil may, indeed, be universal good, and those laws which we cannot reconcile with our limited notions of benevolence and justice, may form the part of a whole system which exhibits the highest degree of both.

This is the manner in which the accusation of partiality and cruelty on the part of the Creator, for inflicting on his creatures physical and moral evil, has been met by some of the ablest theologians. Butler, in that noble work, the Analogy between Natural and Revealed Religion, adopts, if our memory serves us, this defence. Paley does the same. It is we think, in a philosophical and moral sense, a better defence than to justify the infliction of misery and wrong, by what must be considered maxims of state policy. That much unhappiness, not absolutely springing ex necessitate rei, has been permitted to exist, is not and cannot well be denied. That inevitable admission is so far an acknowledgement of a defect, either of unconditional benevolence or unconditional power, in the Creator. But either is a monstrous supposition; and, in the choice of difficulties, it is easier to suppose our faculties too narrow to perceive the adaptation of the actual arrangement to a great scheme of creation, in itself most wise and good, than to conclude that a Deity, who on every side displays consummate art and obvious beneficence, should here, without an object, fail in both. In short, both the religious and the reasonable man must humbly own his ignorance, and deprecate the anger of an Almighty Being, who has shewn that he can punish as well as reward. How splendid is the apostrophe of Pope to the complaining infidel.

> Presumptuous man! the reason would'st thou find,
> Why form'd so weak, so little, and so blind!
> First, if thou canst, the harder reason guess,
> Why form'd no weaker, blinder, and no less?
> Ask of thy mother earth why oaks are made,
> Taller and stronger than the weeds they shade!
> Or ask of yonder argent fields above,
> Why Jove's satellites are less than Jove!

The carnivorous system has, chemically speaking, this effect—it occasions a remarkable similarity of composition in the great family of organized beings. They may be said to be essentially made up of three great staminal principles—the saccharine principle, chiefly found in the vegetable kingdom, and formed of carbon and water—the oleaginous principle, existing both in vegetables and animals, affecting both solid and fluid forms, and composed either of olefiant gas and water, or of some analogous constituents—and finally, the albuminous principle, comprising gelatine and gluten, and displaying in its constitution the superadded element of azote.

Sueh are the three staminal principles of organization;—their forms are infinite—their convertibility considerable—and, under the influence of the vital agents, new principles, proximate principles, of course, appear at times to spring from them. If such be the chemical analysis of organization—if all animals are composed of these three great staminal principles—it must follow that their food, that is their own materials, must necessarily consist of one or more of the principles in question. Such is found to be the fact.

"Hence, it not only follows, as before observed, that in the more perfect animals, all the antecedent labour of preparing these compounds *de novo*, is avoided; but that a diet, to be complete, must contain more or less of all the three staminal principles. Such, at least, must be the diet of the higher classes of animals, and especially of man. It cannot, indeed, be doubted, that many animals have the power of forming a chyle, and, if expressly organized for the purpose, may even live for a while on one of these classes of aliments; but that they can be so nourished for an unlimited time, is exceedingly improbable. Nay, if we judge from what is known from universal observation, as well as from experiments which have been actually made by physiologists regarding food, we are led to the directly opposite conclusion, namely, that the more perfect animals could not so exist; but that a mixture, of two at least, if not of all the three classes of staminal principles, is necessary to form an alimentary compound, well adapted to their use.

This view of the nature of aliments is singularly illustrated and maintained by the familiar instance of the composition of *milk*. All other matters appropriated by animals as food, exist for themselves; or for the use of the vegetable or animal of which they form a constituent part. But milk is designed and prepared by nature expressly as food; and it is the *only material* throughout the range of organization that is so prepared. In milk, therefore, we should expect to find a model of what an alimentary substance ought to be—a kind of prototype, as it were, of nutritious materials in general. Now, every sort of milk that is known, is a mixture of the three staminal principles we have described; that is to say, milk always contains a *saccharine* principle, a *butyraceous* or *oily* principle, and a *caseous*, or strictly speaking, an *albuminous principle*. Though, in the milk of different animals, these three staminal principles exist in endlessly modified forms, and in very different proportions; yet neither of the three is at present known to be entirely wanting in the milk of any animal." 478.

Dr. Prout observes in passing, that the formation, situation, opportune production, and composition of milk, are as clear an instance of design as any in the creation. This is so obvious that we need not dwell on it. To imitate milk, not in colour nor in taste, but in actual chemical constitution, is, according to our author, the great art of cookery, and of the cook: and however indignant M. Ude may feel, this is undoubtedly the fact. The mixture of the three great staminal principles is the end and, unwittingly it may be, the aim, of the thousand combinations of butter, and sugar, and flour, and eggs, that, under tens of thousands of names, issue from the steaming laboratories of the kitchen and the bakehouse. To man, amongst his other privileges, has been accorded that of cookery; yet so bad an use has he made of it, that, instead of displaying gratitude for his power of torturing his aliments into paste, and stews, and fricandeaus, the popular voice has proverbially assigned both cookery and cooks to an origin any thing but divine.

Dr. Prout proceeds to investigate the digestive process. He makes some remarks on the manner in which water enters into the composition of bodies, which merit the attention of our readers.

Water is mixed up with most organized beings in two separate modes, or forms. It may constitute, he says, an *essential* element of a substance, as of sugar or of starch in their dryest states; in which case, the water cannot be disunited without destroying the compounds: or water may constitute an *accidental* ingredient of a substance, as of sugar or of starch in their moist states; in which case more or less of the water may frequently be removed, without destroying the essential properties of the compound. Now, a very large number of organized bodies (perhaps all those to which our present enquiry relates), contain water in both these forms; both as an essential element, and as an accidental ingredient; and in most instances, it is impossible to discriminate between the water that is essential, and that which is accidental. The mode of union, however, among the elements of bodies, in these two states of their combination with water, must be altogether different. The difference is one of those subtle facts, enveloped in the mystery of organic agencies and actions. Dr. Prout offers some very ingenious observations on the laws which appear to regulate it, but, for these, we are compelled to refer our readers to the work itself. We will therefore proceed to the chemical operations of that organ, into which the aliment is introduced—the stomach.

Our readers must all know that very different opinions have been entertained with respect to the operations of the stomach. It has been successively regarded as a mill—a stew-pan—a vat—and, of late, as a galvanic trough. The prevailing science of the day has claimed the stomach as its own, and, the various sects who have reigned in medicine have successively found in the operations of this organ an example of the powers which constituted the Archæus of their doctrines. Some physiologists have run, perhaps, to an opposite extreme, and, disgusted with the attempt to reduce vitality to physics, have claimed for the stomach a superiority to chemical and mechanical operations, as absurd and erroneous as its exclusive degradation to the one or the other. When we see on the one hand an apparatus, palpably calculated for trituration, and pouring out a fluid which exerts a marked chemical action on the food—and when we view, on the other, the effects of numerous vital agencies, as the influence of the mind and appetites, on this same organ of chemical and mechanical construction—we must admit at the very threshold of inquiry, that it would be unphilosophical to seek in any single agency, the solution of its complex functions. We shall probably find that though the means are physical, and obvious, the controlling power is essentially vital and recondite. Dr. Prout offers the following view of the general operations of the stomach.

1. "The stomach has the power of dissolving alimentary substances, or, at least of bringing them to a semifluid state. This operation seems to be altogether chemical; and is probably effected by *reducing* the properties of these alimentary substances.

2. The stomach has, within certain limits, the power of changing into one another, the simple alimentary principles, which have been described in the last chapter. Unless the stomach possessed such a power, that uniformity in the composition of the chyle, which we may imagine to be indispensable to the existence of every animal, could not be preserved. This part of the operations of the stomach, appears, like the reducing process, to be chemical; but not so easy of accomplishment; it may be termed the *converting* operation of the stomach.

3. The stomach must have, within certain limits, the power of *organizing* and *vitalizing* the different alimentary substances; so as to render them fit for being brought into more intimate union with a living body, than the crude aliments can be supposed to be. It is impossible to imagine, that this organizing agency of the stomach can be chemical. This agency is *vital*, and its nature is completely unknown." 489.

The above may be considered a very fair estimate of the action of the stomach—an action, so far as the alterations of the food go, chiefly chemical, but partly vital also. The transcendental vitalists are loth to admit the operation of chemical agents at all, and would seem to consider it derogatory to suppose that any changes, save the subtle ones effected by the powers of life, are worked upon the aliment. Yet in this, as in other instances these gentlemen betray a great ignorance of the mode in which the vital principle acts on matter, and, in their anxiety to avoid materialism, they run into a grosser error still. The vital principle, whatever it may be, incessantly makes use of chemical and mechanical agents for its purposes; and it is no more degrading to it to employ an acid liquid, and a triturating process in order to digest the aliment, than it was to have recourse to bony levers, cartilaginous pulleys, and tendinous ropes, in order to obtain it. There cannot be a more ridiculous or more pernicious error, than the attempt to strain beyond its just limits the immediate operation of the vis vitæ. The science of organization has made its great advances, by endeavouring to limit it, and by viewing the processes carried on in animals and plants, as the results of chemical and of physical operations, the instruments of the vital influence, which presides over them.

Dr. Prout first examines the reducing power of the stomach.

To ascertain in what it consists, he introduces into the stomach an albuminous fluid, and examines the alterations that it undergoes in its conversion into chyme. When received into the stomach, an acid secretion is poured on it, and it is coagulated, just as the albumen would be coagulated by an acid fluid out of the organ. After a time the coagulated albumen becomes softened, and at last it is changed into a nearly fluid chyme. In the chyme, it is still albumen, but its powers of coagulation are much weakened, apparently, from its combination with water. Thus the organic agent reduces the albumen from a strong to a weak condition. It is mixed too, we must remember, with fluids already animalized, the gastric fluid, and the mucus of the stomach.

Cookery assists the stomach, because it is itself a reducing process. Even the woody fibre may be changed, by alternate baking and boiling, into a sort of bread. Dr. Prout laments that cooks are not chemists. Their principal aim appears to be to make unwholesome things pleasant to the palate. Were they chemists, they would, perhaps reverse the proceeding and render wholesome things extremely nasty. In some disordered conditions of the stomach, its reducing or dissolving property is greatly deteriorated, and the power of digesting solid food is for the time destroyed. In some of those forms of indigestion, characterised by excessive sensibility of the nerves of the stomach, even the smallest quantity of fluid farinaceous aliment, is productive of much suffering when received into the stomach, and beyond this the organ can perhaps assimilate nothing.* In other diseases, as in diabetes, and in those extraordinary instances of bulimia that have from time to time been noticed and recorded, the reducing power of the stomach is inordinately increased. Before dismissing the subject we may observe, that the difference in this respect in different individuals is remarkable—some being endowed by nature with stomachs almost as potent as the gizzard of a granivorous bird; and others being afflicted with a natural delicacy of this important organ, a delicacy which is productive of no little misery in life.

It is interesting to inquire by what means the combination of the alimentary substances with water is effected. The gastric juice, is, certainly, the

---

* The curious reader may consult the work of Dr. Johnson, on morbid sensibility of the stomach and bowels.

most important agent in this operation, and it seems to owe its efficacy chiefly to chlorine. If, instead of that element, much free muriatic acid is elicited, great uneasiness, and more or less derangement of the process of digestion follow. Dr. Prout's speculations on the source of the chlorine are too curious to be suffered to escape unnoticed.

"The source of this chlorine or muriatic acid, must be the common salt which exists in the blood: to suppose that it is generated, is quite an unnecessary hypothesis. The chlorine is therefore secreted from the blood; and it may be demanded, what is the nature of the agency, capable of separating that element from a fluid so heterogeneous as the blood? We are acquainted with one agent that exerts such a power, namely, electricity; and this agent, as we formerly observed, seems to be employed by the animal economy for its operations, in the same manner, and on the same principles, as the materials themselves are employed, from which the animal body is constructed. Perhaps, therefore, the decomposition of the salt of the blood may be fairly referred to the immediate agency of this principle, electricity. But here the question arises, What becomes of the soda from which the muriatic acid has been disunited? The soda remains behind, of course, in the blood, and a portion of it, no doubt, is requisite to preserve the weak alkaline condition essential to the fluidity of the blood. But the larger part of this soda is probably directed to the liver, and is elicited with the bile in the duodenum, where it is thus again brought into union with the acid, that had been separated from the blood, by the stomach. These observations, illustrating the importance of common salt in the animal economy, seem to explain, in a satisfactory manner, that instinctive craving after this substance, which is shewn by all animals.

Admitting that the decomposition of the salt of the blood is owing to the immediate agency of galvanism; we have, in the principal digestive organs, a kind of galvanic apparatus, of which the mucous membrane of the stomach, and perhaps that of the intestinal canal generally, may be considered as the acid or positive pole; while the hepatic system may, on the same view, be considered as the alkaline or negative pole. Whether such galvanic action be admitted or not; and the admission is of no very great importance; what we have above stated may be received as a simple expression of the facts, in so far as they relate to the saline constituents of the blood. Moreover, be the nature of the energies what they may, by which these changes are effected; along with these changes, and probably by the aid of the same energies, other very important changes or processes are carried on, to some of which we shall presently have occasion to allude. In the mean time, we may close this section by observing that there is strong reason to believe, that the solvent power, which we have described, or some power having a great resemblance to it, exists not only in the stomach, but in every part of an animal body. In all animals there are minute tubes, called absorbents, which originate in every part of their bodies, and at length uniting, enter the sanguiferous system along with the chyle. Now, the office of these tubes, is to remove all those portions of the animal frame, which after having performed their several functions, require to be withdrawn. Of course, before solid parts can be thus removed, they must be dissolved, (*digested* in fact); and such solution, in many instances, is probably effected, as it is in digestion, by combining these solid parts with water. This supposed analogy between the solvent powers of the stomach, and those which must prevail all over the body, seems to be strongly confirmed by that similarity of structure and of function existing between the lacteals and the absorbents: they indeed form but one system." 498.

We see no great objection to supposing that the vital principle makes use of galvanism to separate the chlorine and the soda from the blood, but neither do we see any proof of its doing so. There is no reason for withdrawing the secretion of the gastric juice and of the bile, of the chlorine in the former and the soda in the latter, from under the control of the laws and the powers that regulate secretion in general. If that be galvanism, the nervous influence, for to the nerves the dominion of secretion is confided, is galvanism. In spite of the positiveness of Dr. Wilson Philip, this is yet unproved, and, probably, in the present condition of our knowledge, the most philosophical conclusion, is to look upon the nervous power as essentially vital and organic. It is possible, yet we scarcely conceive that it is likely, that the progress of physical inquiry may ultimately resolve this and other agencies that we now deem vital, into those which regulate matter in the gross. That period has not yet arrived, and we are bound to reason in accordance with the facts that we do know.

It appears to us that the analogy between the stomach and the absorbents of the body, is not so close as, on a slight examination, it may seem. The stomach pours out an acid liquid, which acts on a heterogeneous mass, and converts into a homogeneous paste. Nothing of the sort is proved in regard to the absorbents. They take up the fluids and the solids of the body, but, so far as we know, they effect little change in either. To compare them to the lacteals, is a different thing from comparing them to the stomach, for the lacteals appear to do little more than simply absorb, and, perhaps, select the matters for absorption. If the absorbents are analogous to the stomach, the veins are so too, for their absorbing powers are now undoubted. The truth is, that we are very imperfectly acquainted with the mechanism or the chemistry of either venous or lymphatic absorption.

2. The converting power of the stomach, consists in its altering the disposition of the ultimate elements, in the proximate organic principles submitted to it. As the chyle is very uniform in composition and consists essentially of albuminous and oleaginous materials, and as a large proportion of the food of most animals is made up of saccharine matters, the conversion of this into the oleaginous and albuminous principle must be effected. How it is so we do not know, nor can we explain the origin of the azote in the albumen. It is certain that, out of the body, sugar is convertible into alcohol, and alcohol is a weak oleaginous substance. The adaptation of the latter to the purposes of nutrition is sufficiently notorious. Hybernating animals are enormously fat when they retire for the Winter, but during their hybernation, all the adeps is removed.

3. The organizing and vitalizing powers of the stomach may be summarily disposed of. We are ignorant alike of their nature and their mode of operation. We here arrive at the ultimate facts of vitality, and our reason is unable to penetrate its arcana.

The changes that the food undergoes in the duodenum, are familiar. The bile and the pancreatic juice are poured upon it, and the chyle begins to be distinctly separated from those matters which are finally to be voided in the shape of excrement.

The composition of the bile, which is wonderfully similar in all animals, may be broadly stated as consisting chiefly of water and of various solid matters in which carbon and hydrogen predominate. These exist in conjunction with soda and its salts, and by the latter the bile is rendered alkaline.

The pancreatic secretion contains some albumen and a curdy substance, holds some saline matters in solution, and is slightly acid.

The changes that the chyme submits to in the duodenum may be briefly stated. From being acid, it grows neutral, or almost neutral—if no albumen was before perceptible it now becomes so—a little gas is extricated—and the separation into chyle and excrementitious portions begins. Such are the more obvious and remarkably constant alterations of the food effected in the duodenum. The process of vitalization that goes on there, baffles our means of enquiry and of comprehension.

In the alimentary canal, beyond the duodenum, the chyle is fully formed and absorbed, the excrement fully separated and removed.

Dr. Prout concludes the chapter by a few very interesting and judicious observations, on the nature of food and on its mode of preparation. He remarks that a knowledge of the process of digestion teaches us that the less organized our aliment is, the less it is digestible. Crystallized, or very pure substances, are consequently difficult of assimilation. Pure sugar, pure alcohol, pure oil, are not so easily digested as amylaceous matters, wines, or butter. Of all crystallized matters, pure sugar would seem to be assimilated with most facility, yet often it is utterly pernicious, and it seldom or never can be considered wholesome. It is man who has manufactured pure sugar and pure starch, and in this, as in other instances, he has shewn himself over-officious. Intent on gratifying the senses, he has wandered from the salutary simplicity of Nature, and the modern voluptuary, who sits daily at the board which has been decked by Ude, should reflect that the oil, and the sugar, and albumen which perverted cookery has tricked out in such unnatural and alluring forms, are poured into a stomach unequal to this incessant demand upon its energies. Gout, and gravel, and plethora, in its many consequences, wait upon the gourmand, and a sickly offspring will inherit his impaired digestion, perhaps his vicious tastes. Philosophy prescribes, but how seldom will wealth and sensuality take, a simpler diet and less luxurious cookery. If the dishes of France are perniciously complicated, Dr. Prout is of opinion that our own are too crude, and that here sufficient attention is not paid to the process of culinary reduction. Certainly our puddings and our pastry are execrable, and require for their assimilation all the vigour of a stomach fed with our plain animal food and strong wines. Yet it cannot be denied that, whatever faults may attach to our national diet, it is more calculated to give birth to bodily vigour, than the greasy compounds which our Continental neighbours indulge in.

In the following sentiment, we quite coincide with Dr. Prout. We think that the chemical view he has offered of the process of digestion is well calculated to give us a philosophical idea of it, and to substitute an exact simplicity for what was before invested with too much of the obscure and marvellous.

"The view we have now taken of the processes of digestion, removes in some degree that mysterious character with which they have been invested; and by lessening the field of our enquiry, brings us nearer to our object. We had previously known, that the articles employed as food by animals are essentially composed of three or four elements. But we have now learnt, that all the more perfect of those matters on which animals subsist, are compounds of three or four proximate principles; the whole of which compounds, except one, are, in their essential characters, identical with those composing the frame of the animals themselves. We have also learnt, that owing to this identity of composition, many animals are saved the labour of forming these proximate principles from their elements; and have only to re-arrange them, as their exigencies may require. The task of forming the proximate principles is thus left to the inferior animals or to plants; which are endowed with the capacity of compounding them from matters still lower than themselves, in the scale of organization. Hence there is a series, from the lowest being that derives its nourishment from carbon and carbonic acid, up to the most perfect animal existing. Each individual in the series preferring to assimilate those immediately below itself; but having, on extraordinary occasions, and in a minor degree, the power of assimilating all, not only below, but above itself, in the system of organized creation." 511.

Setting aside all considerations of the question of animals being doomed to prey upon each other, and taking the carnivorous system as it is, the perfection of that system is very admirable. One creature works unconsciously, yet with perfect regularity, for the other, and each animal and plant feeds on some part of the organic world, and becomes the food for others in its turn. Man appropriates the labours of all, and, surrounding himself with the spoils of innumerable victims—ministering, with the destruction of other living things, to every want and every wish—straining his intellect to discover new sources of sensual indulgences, to be gratified still at the expense of the animal creation—he complacently sits down, and questions the benevolence of a Deity who permits a tiger or a shark. What mockery is this! The atheist has been endowed by a kind Creator with reason, which may enable him to shun the perpetration of unnecessary cruelty, and to raise himself, if he pleases, above the level of the beings born to yield to an indomitable instinct. Yet too often the discourser on the savageness of nature evinces no intention of dispensing with its privileges, and tickling his palate with mollusca when in season, he continues his elaborate and eloquent invectives against carnivorous feeding.

The consummate art with which the matters least calculated for affording nutriment to the higher classes of animals have still been made indirectly to sustain them, has often been a theme of admiration. The grasses, for example, will not by any cookery support the life of man. Yet the sheep and the bullock, gifted with a most elaborate contrivance for extracting their nutritious elements, have formed in all ages man's principal food. In this oblique manner, man may be said to feed upon the grasses. It would occupy us too long to point out the necessity for enabling him thus to make use of plants, the most widely diffused and most easily produced. Had the animals on whom he must chiefly feed, been unable to subsist on so common and prevalent a species of nutriment, man must have been incessantly in danger of starvation. On the whole, it is impossible not to perceive the adaptation of things in the organs of digestion and the aliment—it is impossible to deny the evidence of elaborate design, consummate wisdom, and infinite benevolence.

The fourth chapter, the last, is occupied with the conversion of chyle into blood—with respiration—secretion—the final decomposition of organized bodies—and with some general reflections, which form the conclusion of the volume.

1. There is little to be said on the absorption of the chyle. But Dr. Prout adverts, and we think very justly, to the functions of the absorbents of the body. An opinion has prevailed, that the matters they contain are excrementitious, the debris of the machine, taken up by these sewers and carried off. As Dr. Prout observes, it is not likely that merely excrementitious matter would be mixed with the new chyle, which has just been separated from excrement of another sort. On the contrary, there are many reasons for supposing that, though the lymph contained in the absorbents must be in some degree excrementitious, it is decidedly recrementitious also. Mixing with the new chyle, it tends to effect its animalization, and perhaps, as Dr. Prout remarks, the lymph is destined to undergo repeated organizing processes.

2. The blood, into which the chyle and lymph are poured together, is essentially, as all our readers know, an albuminous fluid. Now it is a fact, that *gelatine* is never found in the blood, nor in any product of glandular secretion. Dr. Prout observes that gelatine appears to rank lower than albumen in the scale of organized substances, and that a given weight of it contains three or four per cent. less carbon, than an equal weight of albumen. The production of gelatine must therefore be a reducing process.

3. Passing over the mode in which the blood is conveyed to the lungs, we may pause at the last chemical theory of respiration, and of the gazeous changes in the air, that are observed in it.

"On examining the respired air, a remarkable alteration of its properties is found to have taken place; a portion of its oxygen has disappeared, and a similar bulk of carbonic acid gas has been substituted. With respect to the origin of this carbonic acid gas, there have been various opinions. Formerly, the greater number of physiologists maintained that carbon, in some form, was excreted by the lungs; and that this excreted carbon, uniting with the oxygen of the inspired air, was converted into carbonic acid gas. No one imagined that oxygen gas could be passed inwards through the membrane of the lungs, while carbonic acid gas was, at the same time, passing outwards, through the same membrane. Accurate observations have, however, demonstrated, that such a simultaneous passage of gases really takes place through the membrane of the lungs; and the observations are not confined to the two gaseous bodies in the lungs; but are applicable to all gases whatever, under similar circumstances. In consequence of these observations, it seems now to be generally admitted, that the oxygen of the atmospheric air is absorbed by the blood, and, in some unknown state of combination, reaches the extreme subdivisions of the arteries; where it is united with a portion of carbon, and forms carbonic acid gas: that this carbonic acid gas is retained in some unknown state of combination in the venous blood; till, in the lungs, it is expelled, and oxygen is absorbed in its stead; according to the laws which regulate the diffusion of gaseous bodies, formerly explained. Further, with the carbonic acid gas, a large quantity of aqueous vapour, as we have stated, is at the same time separated." 523.

The bright colour of arterial blood, which was formerly considered exclusively due to oxygen absorbed, would seem to be much influenced by the saline matters contained in the blood; the dark colour of venous blood is chiefly owing to the presence of carbonic acid gas.

"What is the source (says our author) of the carbonic acid in venous blood, and of the aqueous vapour that is expelled from the lungs? These questions cannot be answered with certainty. But some observations lately made, have induced us to believe, that the *conversion of albuminous matters into gelatine*, is one great source of the carbonic acid in venous blood. Gelatine, which, as before observed, contains three or four per cent. less of carbon than albumen contains, enters into the structure of every part of the animal frame, and especially of the skin: the skin indeed consists of little else besides gelatine: it is most probable, therefore, that a large part of the carbonic acid of venous blood is formed in the skin, and in the analogous textures. Indeed, we know that the skin of many animals gives off carbonic acid, and absorbs oxygen; in other words, performs all the offices of the lungs; a function of the skin perfectly intelligible, on the supposition that near the surface of the body, the albuminous portions of the blood are always converted into gelatine. With respect to the aqueous vapour thrown off from the lungs: we have every reason to believe, as before stated, that much of this vapour is *derived from the chyle*, in its passage through these organs; and that by such separation of water, the *weak* and delicate albumen of the chyle is converted into the *strong* and perfect albumen of the blood; according to the principles detailed at the commencement of this chapter." 525.

The precise use of the constant evolution of carbon, as well as the mode in which it is effected, are points involved in much obscurity. It is probable that, whatever other causes of animal heat there may be, the production of carbonic acid gas in the body is one. Why so much carbon should be extricated from the body as there actually seems to be, is by no means obvious.*

There can be no doubt that the extrication of carbonic acid by animals, is subservient to the support of plants, of which it forms the principal food. We now see, says our author, that nearly the whole of the superfluous carbon produced by the operations of the animal system, is actually thrown off in the form of carbonic acid. Plants, therefore, on the one hand, supply the chief nourishment to animals; while that gaseous matter which is separated by the animal economy, and which, if retained within animals, would to them be fatal, constitutes, on the other hand, the chief food of plants. Nor, in these two respects only, are the two great systems of organization mutually dependent; for unless plants consumed the carbonic acid gas which is formed by animals, this deleterious compound would probably accumulate in the atmosphere, so as to destroy animal life; while it is doubtful whether the present races of vegetables could exist, if carbonic acid gas were not formed by animals.

We think it is scarcely necessary to pursue the argument of design. Wherever we search, we discover nothing else. In one instance, perhaps, the evidence is more distinct, the contrivance more palpable and gross, the case better suited for a mere argument; but design is every where apparent, at least to him who looks, not predetermined to be blind.

Dr. Prout very properly directs attention to the adaptation of mechanical contrivance to chemical operations in the economy—an adaptation which conclusively establishes design.

"Thus, when we witness such a display of elaborate arrangements as are exhibited in the mechanism of the digestive organs, and of the circulating system; the purpose of which arrangements is merely to produce a few chemical changes in the food, and in the blood; it is evident that the chemical changes so pro-

* It has been calculated, perhaps extravagantly, that the lungs of a man of ordinary size expel eleven ounces of carbon in the course of twenty-four hours.

duced, must be at least as real as the mechanical structure, by means of which they are effected. Hence the adaptations of mechanical arrangements, in the structure of organized beings, to the pre-existing chemical properties of matter, affords an evidence of design, not less impressive than unequivocal. The most determined sceptic cannot assert that there is any *necessary* relation, or indeed any relation whatever, between the mechanical arrangements, and the chemical properties to which they administer. There is no reason why the chemical changes of organization should result from the mechanical arrangements by which they are accomplished: neither is there the slightest reason why the mechanical arrangements, in the formation of organized beings, should lead to the chemical changes of which they are the instruments. From what cause, then, arose the association of the chemical changes with the mechanical arrangements? How were the chemical operations of digestion and of respiration brought into union with the mechanical apparatus of digestion, and with the circulating system? The co-existence of things so entirely dissimilar, and having no kind of mutual relation, can be explained only on the supposition that *a will exists somewhere;* and also a *power* to *execute that will.*" 541.

All organic bodies, admirably contrived as they have been, are doomed to exist as living things but for a season. All nature is in motion, all organism is a cycle. Birth, growth, maturity, decline, and death, are the destiny of animal and plant. But organic death is not even material annihilation, for the gases and the elements of which the corporeal fabric was constructed, when liberated by the process of decomposition from *it*, are speedily engaged in ministering to the constitution of some other organized being. The Pythagorean applied to the soul, the laws which modern chemistry has shewn to regulate the body. If our spirit does not transmigrate, our elementary components do, and "the noble dust of Alexander," which Hamlet conjectured might be "stopping a bung-hole," was perhaps, at that instant, forming a part of the thinking brain of the Dane himself.

The dissatisfied sceptic, doubting if he was created, repines that he is to die. Eccentric reason generates the infidelity—Nature dictates the complaint. It does, indeed, seem a hard decree, which has gone forth against the sons of men:

The race
Which could not keep in Eden their high place,
But listened to the voice of knowledge without power,
Are nigh the hour
Of death.

The pride of humanity would be gratified at the consciousness, that *it* was exempt from the law of change that governs the rest of the Creation. That law is, doubtless, the result of the same consummate wisdom that is evident in every thing besides. Constituted as we are, the change at which we murmur is the source of very much of our happiness, and of most of our improvement. In political life, it prevents the despotism into which institutions would converge—in moral life, it uproots the vices which infest old nations—and in the scheme of nature it maintains perpetual youth. Had we been created with other aptitudes, and surrounded with other circumstances, the same Omnipotence that has fitted us for the world we actually exist in, would have equally fitted us for that. Probably, in the planets we distinguish, and in those that we cannot, different forms and laws of life may include some approach to individual perpetuity. It is certain that we form but an atom in the Universe, and what blasphemous folly it is in us, to erect our ignorance as the tribunal for the arraigning of our God.

In the last few pages of the treatise from which we are shortly to part, Dr. Prout offers some admirable reflections on the province and the prospects of chemistry. It always gives us the highest pleasure to enforce what we consider philosophical habits of thought. Amongst the readers of this, as of all medical journals, there must necessarily be many of imperfect education, of limited opportunities, whose habits of observation were never directed by an acquaintance with the higher laws of reasoning, and whose conclusions are not likely to spring from very exact investigation, or to be regulated by a very philosophical spirit. We regret to perceive in few of our medical works the disposition to imbue the mass of the profession with the spirit we allude to. We commonly distinguish little of that elevated sentiment, which, raising itself above its immediate object, aspires to inculcate the more intellectual habits of action and thought. The pages of our periodicals, too, are often occupied with the passions and the prejudices of the day; and even if party considerations are disregarded, the critic seldom rises above the mediocrity of the occasion. Were there nothing to correct this in medical periodical literature, it would be a subject of serious regret. But we trust that, with the advance of literature and of science, a higher tone of professional criticism will prevail, and that medical journals will not be behind those devoted to the diffusion of general knowledge. Authors who feel the severity of criticism, are too apt to impute it to any cause but the real one, and to construe into personal attacks, and attribute to personal feelings, remarks that have their origin in the plan of conduct and temper of mind to which we have alluded. But, if the critic is honest, his intentions, in the long run, will not be misconceived, and temporary misrepresentation is of little moment. But to return to the prospects of animal chemistry.

Chemistry must be considered intermediate between a science of quantity, and one of exclusive experience. Any science of quantity is, of course, demonstrative—one of experience is empirical—and, in proportion as any branch of knowledge approaches to the one or to the other, it acquires the peculiar character of either.

As demonstrative science is most certain and satisfactory, the more demonstrative a science can be made the better.

In the case of organic chemistry, the more we can bring it under the dominion of the laws of quantity, the more fixed our results will be, and the more we shall be enabled to reason from results obtained, to those which are to come.

For, a science of quantity has this advantage over one which is empirical, that we can calculate results beforehand, and, from known premises, obtain yet unknown consequences. Hence the splendid effects of analysis in the hands of the modern French algebrästs.

Physiology embraces facts and phenomena. The facts are to a certain degree quantitative—to a still greater degree experimental. The phenomena are almost exclusively vital, and not determinable by quantitative laws. The application of chemistry to physiological investigations has done very much for the increase of our knowledge; for it has tended to separate the quantitative from the experimental facts, and it has given to the latter as much certainty as, from the nature of things, they will admit of.

We say as much certainty as they will admit of. For observe the different circumstances in which the pursuit of positive and of experimental knowledge is placed. Quantitative facts once made out, are susceptible at any time of confirmation. But experimental facts are ascertained by observation, and only supported by testimony. The observation may be imperfect, or absolutely false, the testimony may also be one or the other. Admission of such facts is dangerous—their refutation is troublesome, and may not be possible, because, as experiment presupposes opportunity and concurrent circumstances, such opportunity and circumstances may be inaccessible. And this is the cause of the disputes, the dogmas, the revolutions that have distinguished physiology hitherto, and which, in some measure, must continue to infest it.

It is, therefore, an object of the highest moment to apply chemistry, as much as possible, to the investigation of the nature of organic bodies. What quantitative, what exact knowledge is possible, has been and will be chiefly obtained through its means. But two things are requisite on the part of the chemical enquirer—accurate observation, and fidelity of report. We cannot do better than terminate these remarks with the following passage from our author.

"The progress that chemistry, within these few years, has made, is truly astonishing; and when a more rigorous mode of observation shall be adopted—in short, when chemistry shall be brought more under the control of the laws of quantity—a control that will be exercised, at least indirectly—it is impossible to foretell the degree of perfection which chemistry, as a science, may attain. But, for many years yet to come, the progress of chemistry must depend solely on experiment; and its cultivators must be satisfied with the comparatively humble office, of discovering the actual chemical changes which bodies effect on each other.

Since, then, in knowledge derived from observation, an acquaintance with *what exists* and with *what is done*, is indispensable, to obtain a clear, accurate, and unequivocal conception of these things, is the first duty of every observer, and of every experimentalist. Nor is there any observer or experimentalist, however unpretending, who may not add to the stock of ascertained facts; so varied and inexhaustible are the stores of nature. Another duty of every one who engages in observation or experiment, is to become the faithful historian of what he witnesses; to narrate the event or phenomenon in plain and intelligible language, employing only terms of a definite meaning, so as to convey to others a just notion of what he has seen. We say a *just* notion; in the greater number of instances, a *perfect* notion is impossible, because what is seen cannot be expressed by language. But to give a just notion; that is to say, a notion which, though incomplete, *has no foreign or false gloss,* is within the power of every observer: and to give such a notion of the facts he narrates, ought to be his chief study. One testimony of so faithful a witness is often invaluable, and worth a thousand vague and inaccurate observations, which are only calculated to bewilder or to mislead, and thus are worse than useless.

The next rule which an interpreter of nature should bear in mind, is *not to attempt too much at first;* but in order to establish a sure foundation for his succeeding labours, he ought to be content with obvious and unexceptionable facts, and so to arrange these facts as to lead to others. To elicit novel and prominent facts is the lot of few; and any one may happen to be so successful. But all, as before stated, may *investigate truth;* and thus contribute more or less towards the advancement of knowledge. Moreover, the humblest contributors may rest assured, that they are imperceptibly raising a structure, which will sooner or later include the conspicuous labours of their more fortunate coadjutors; in which structure, these labours will indeed still appear conspicuous, though their importance will be diminished as the fabric is extended around them." 548.

With this quotation we conclude. We have derived much pleasure, and not a little information, from travelling through the organic world with so instructive and amiable a companion. The labours of Dr. Prout will be every day more fully appreciated by the profession—labours which have done much to philosophize our acquaintance with those departments of organic chemistry, and of pathology, to the elucidation of which they have been directed.

